# Masturbatory Guilt Leading to Severe Depression

**DOI:** 10.7759/cureus.13626

**Published:** 2021-03-01

**Authors:** Yahia Albobali, Mahmoud Y Madi

**Affiliations:** 1 Psychiatry, Hamad Medical Corporation, Doha, QAT; 2 Internal Medicine, The University of Kansas Medical Center, Kansas City, USA

**Keywords:** depression, adolescents with psychiatric disorders, sexual behaviour

## Abstract

Masturbation is a common sexual behavior in humans. However, it is viewed negatively across cultures and is prohibited by almost all religions. These views lead to certain cultural beliefs that affect the sexual behavior of people and have implications on the mental health of the individual. There is limited literature linking masturbatory guilt with psychopathology. Here, we present a case in which masturbatory guilt contributed to the development of a depressive illness in a young male. The patient presented with typical symptoms of severe major depressive disorder with resulting impairment of academic and social performance. Our approach to treatment included utilizing a combination of psychotherapy, antidepressant and antipsychotic medications and vitamin supplementation with notable clinical improvement. The article highlights the importance of incorporating various cultural beliefs into an individualized treatment plan, particularly in unique cases where behaviors that may be stigmatized - perhaps wrongfully so - are involved.

## Introduction

Masturbation is a sexual act that is common in all cultures and throughout history [1]. It is a common behavior among both males and females [2,3]. Despite being a prevalent sexual activity, masturbation is largely viewed negatively and almost all major religions prohibit it [4]. These negative cultural and religious views towards masturbation have generated certain beliefs and myths that affect an individual’s sexual life. This results in certain clinical implications on the mental health of certain individuals. Dhat syndrome, for example, is a culture-bound syndrome seen in the Indian subcontinent, which is characterized by fatigue, weakness, loss of appetite, anxiety and guilty feelings attributed to semen loss through masturbation or nocturnal emission. In this syndrome, the mental health pathologies include depression, anxiety and hypochondriasis. It is proposed that these symptoms stem from the individual's fear of semen loss [5]. Shen-kui syndrome is a Chinese culture-bound syndrome with physical and psychological symptoms (weakness, dizziness, musculoskeletal pain, high levels of anxiety and fear) attributed to excessive loss of semen, which leads to an imbalance in Yin and Yang [6]. There is scant literature about the link between masturbatory guilt and severe psychopathology. Aneja et al. reported three cases in which masturbatory guilt was associated with severe psychopathology, two of the cases presented with depressive disorder and one with psychosis and in two of the cases the masturbatory guilt was in the form of delusional belief [7]. There is also a case report in which the authors reported a 23-year-old man presenting with severe depression and erectile dysfunction associated with masturbatory guilt [8]. In this report, we present the case of a 17-year-old young man who presented with severe depression in the context of masturbatory guilt.

## Case presentation

A 17-year-old single Muslim young man presented to the outpatient psychiatry clinic with three months history of deterioration in his mental and physical health attributed by the patient to a few months period of daily masturbation that preceded a constellation of symptoms including depressed mood, “inability to feel anything anymore” and losing the pleasure in activities that he used to enjoy like watching movies or playing online games. He also described poor memory and concentration, which was particularly significant to the patient as he was in his last year of high school. This resulted in the loss of ability to study properly and decreased academic performance. He had interrupted and unrefreshing sleep, poor appetite and loss of taste. He had significant guilt as he was convinced that what was happening with him was due to daily masturbation habit. Despite his efforts and attempt to completely stop his daily habit, his symptoms persisted. He felt hopeless about his situation and reported not being able to achieve any of his academic or social goals anymore. He had suicidal ideas as he was hopeless, but he did not have the intention or plan to commit suicide as suicide is prohibited in his religion. In addition, the patient reported having frequent headaches, decreased energy level, and paresthesia in his arms and legs. Of note, the patient reported decreased sexual desire but continued to have a normal erection. The patient felt that he caused “an irreparable damage” to his body. The patient had no history of medical or psychiatric diseases, had no family history of psychiatric illness, and did not have a history of alcohol or illicit substance use.

On mental state examination, he was a young man, tall with a BMI of 23, presented with acceptable grooming and hygiene, had very poor eye contact, and appeared restless. His speech was coherent and relevant with normal rate, tone and volume. The mood was depressed with anxious affect. No hallucinations or hallucinatory behaviors. No persecutory or grandiose delusions were noted. He did not have active suicidal ideation, intentions or plans at the time of the initial interview. He was conscious, alert and oriented. His vital signs were all within normal limits. His basic blood investigations including complete blood count, complete metabolic panel, thyroid hormone levels and urine drug screen were within normal limits except for mildly low levels in vitamin D of 22 ng/mL and vitamin B12 level of 199 pg/mL.

Differential diagnosis included major depressive disorder related to other medical condition. This was ruled out by comprehensive history taking and physical examination compounded with routine lab work as discussed above. Drug and illicit substance use as a cause of the patient's presentation was ruled out by the presented history above and the negative urine drug screen. Bipolar disorder manifesting with the depressive episode was excluded by the absence of prior hypomanic or manic symptoms and by the positive response to antidepressants.

Initially, the patient was treated with fluoxetine 20 mg daily that was increased to 40 mg daily after few weeks. As the patient did not show much improvement after four weeks of fluoxetine, and he continued to experience poor sleep and appetite, 1 mg of daily risperidone was added. The patient was also given the following supplements: Vitamin D2 50,000 units weekly for eight weeks, vitamin B complex daily supplement, and omega-3 tablets 1,000 mg twice daily. Additionally, the patient was referred to psychotherapy for weekly sessions. After three months of combined medications, therapy and supplements, the patient started showing improvement in his overall symptoms, especially his mood, memory and concentration. The improvement manifested by the patient’s ability to pass his high school exams with a satisfactory score. The patient rated his improvement at 90% compared to his initial presentation. Eventually, risperidone was stopped and the patient continued showing sustained improvement.

## Discussion

In the medieval and early modern ages, moralists and theologians condemned masturbation as “a sin against nature”. It was in the early 18th century when Onania (or The Heinous Sin of Self Pollution) was published that moral and religious concerns were combined with the accounts on the detrimental effects of masturbation on physical health [9]. In Onania, young male readers were advised to stop masturbation, threatened with suffering from impotence and being ridiculed by women. Later in the 1700s, connections started to be made between masturbation and insanity, and by the early 1800s, physicians in Europe and America believed that masturbation can lead to insanity [10]. Sigmund Freud believed that there is a connection between masturbation and the development of neurasthenia [11].

In Islam, sexuality is closely integrated with religious rules [12]. Masturbation is considered by most Muslim scholars forbidden. In Quran, there are multiple verses that address sexuality, and although not directly mentioning the act of masturbation, it is understood contextually that it is forbidden. Masturbation is understood as a way leading to adultery so it is forbidden [13]. In Chapter 23, Surah Al Mu’minun, verses 5-7: "And they who guard their private parts, except from their wives or those their right hands possess, for indeed, they will not be blamed; but whoever seeks beyond that [in sexual gratification], then those are the transgressors". Here, any form of sexual gratification outside marriage is considered a transgression of God’s law [14]. A study done in the Netherlands explored the views of Muslim adolescents on sexuality and concluded that Muslim adolescents agreed that masturbation is forbidden, as any other form of sexual activity outside marriage, and they argued that masturbation can lead to more sins [15].

Our patient presented with symptoms of major depressive disorder (low mood, diminished pleasure in activities, decreased appetite, insomnia, loss of energy, diminished ability to concentrate, hopelessness and helplessness feelings, and recurrent suicidal ideation). He also had significant guilt that reached a delusional level. In fact, his guilt was related to two factors: first due to believing that masturbation ruined his life, and second due to believing that he was doing a prohibited act in Islam. Even after significant improvement in symptoms and overall function, he continued to hold the belief about the causal relationship between masturbation and his illness. Given the patient's presentation with depression associated with delusional guilt, a psychotic feature, his depression was classified as severe.

Our treatment approach was multidisciplinary, covering biological and psychological interventions, in addition to nutritional and lifestyle modifications. Combining pharmacotherapy and psychotherapy is superior to using medications alone in acute phase treatment and in maintenance [16]. In addition, there is evidence that low levels of vitamin B12 are associated with depression and that higher levels are associated with better treatment outcomes [17]. Furthermore, there is evidence that omega-3 polyunsaturated fatty acids have a beneficial effect on depression [18].

Physicians may face certain difficulties in counselling patients about their masturbatory behavior, especially if they have masturbatory guilt, as the evidence in the literature shows mixed results in terms of benefits and risks of masturbation. While it is reported that masturbation is healthy behavior, that can improve the mood, develop sexual interests and even reduce prostate cancer risk [19], other studies have shown that excessive masturbation is associated with depressive symptoms, anxious attachment, impaired sexual function in men and women, and greater dissatisfaction with relationships [20]. Taking additional cultural views on masturbation into account, it is difficult to give definite advice to patients on whether masturbation is good for them or not.

## Conclusions

There is a lack of literature on the effects of masturbatory guilt, and the beliefs around masturbation in general, on psychopathology. This area needs further research studies taking into account the different religious and cultural backgrounds of patients presenting with such symptoms.
